# Searching for Potential Lipid Biomarkers of Parkinson’s Disease in Parkin-Mutant Human Skin Fibroblasts by HILIC-ESI-MS/MS: Preliminary Findings

**DOI:** 10.3390/ijms20133341

**Published:** 2019-07-07

**Authors:** Cosima D. Calvano, Giovanni Ventura, Anna Maria M. Sardanelli, Laura Savino, Ilario Losito, Giuseppe De Michele, Francesco Palmisano, Tommaso R. I. Cataldi

**Affiliations:** 1 Dipartimento di Chimica, Università degli Studi di Bari Aldo Moro, via Orabona 4, 70126 Bari, Italy; 2 Centro Interdipartimentale SMART, Università degli Studi di Bari Aldo Moro, via Orabona 4, 70126 Bari, Italy; 3 Department of Basic Medical Sciences, Neurosciences and Sense Organs, University of Bari “Aldo Moro”, 70100 Bari, Italy; 4 Department of Medicine, Campus Bio-Medico University of Rome, 00128 Roma, Italy; 5 Department of Neurosciences, Reproductive and Odontostomatological Sciences, University of Naples Federico II, 80131 Naples, Italy

**Keywords:** biomarker, HILIC-tandem MS, lipidomics, human skin fibroblasts, Parkinson’s disease

## Abstract

Early diagnosis of neural changes causing cerebral impairment is critical for proposing preventive therapies for Parkinson’s disease (PD). Biomarkers currently available cannot be informative of PD onset since they are characterized by analysing post-mortem tissues from patients with severe degeneration of the substantia nigra. Skin fibroblasts (SF) are now recognized as a useful model of primary human cells, capable of reflecting the chronological and biological aging of the subjects. Here a lipidomic study of easily accessible primary SF is presented, based on hydrophilic interaction liquid chromatography coupled to electrospray ionization and mass spectrometry (HILIC/ESI-MS). Phospholipids (PL) from dermal fibroblasts of five PD patients with different parkin mutations and healthy control SF were characterized by single and tandem MS measurements using a hybrid quadrupole-Orbitrap and a linear ion trap mass analysers. The proposed approach enabled the identification of more than 360 PL. Univariate statistical analyses highlight abnormality of PL metabolism in the PD group, suggesting down- or up-regulation of certain species according to the extent of disease progression. These findings, although preliminary, suggest that the phospholipidome of human SF represents a source of potential biomarkers for the early diagnosis of PD. The dysregulation of ethanolamine plasmalogens in the circulatory system, especially those containing polyunsaturated fatty acids (PUFA), might be likely associated with neurodegeneration.

## 1. Introduction

Parkinson’s disease (PD) is a progressively debilitating neurodegenerative disorder, involving several motor functions, vegetative, behavioural and cognitive impairments [1,2,3]. A recent systematic study estimated the prevalence of PD to be approximately 6.2 million people worldwide but the figure may be significantly higher as many people go undiagnosed [4]. As the incidence of this disorder rises significantly with age, and people are living longer, its prevalence is set to rise to nearly 13 million people by 2040 [5]. PD is typically associated to specific loss of dopaminergic neurons in the substantia nigra (SN) and accumulation in Lewy bodies of misfolded proteins composed of aggregated proteins, mainly α-synuclein (α-syn), and other metabolites including lipids [6,7]. The clinical diagnosis of PD is classically based on the examination of motor symptoms which do not manifest until >50% of neurons are lost and rating scales (such as Hoehn and Yahr) are typically used to measure disease progression [8]. Neuroprotective therapies contemplating the use of dopamine precursors (e.g., levodopa) alone or combined with other drugs [9] provide only symptomatic relief but the progression of disease remains, suggesting the involvement of other biological mechanisms.

Currently, there are no effective therapeutic targets or early markers to effectively slow down the progression of PD. Genetic studies of the last two decades have identified several genes associated with familial PD. In particular, autosomal-dominant PD includes genes as *PARK1*, *PARK4*, *PARK5*, *PARK8*, *PARK11*, *GIGYF2,* and *PARK13* which code for α-syn, ubiquitin C-Terminal Hydrolase (*UCHL1*), and leucine-rich repeat kinase 2 (*LRRK2*) while autosomal-recessive PD involves genes *PARK2*, *PARK6*, *PARK7* and *PARK9* which code for parkin (PRKN), phosphatase and tensin homolog (*PTEN*)-induced putative kinase 1 (*PINK1*), deglycase *DJ1* and *ATP13A2* [10,11,12]. Some of these studies showed a strong association between PD and mutations in mitochondrial DNA [13], pointing out that mitochondrial respiratory disorder in familiar parkinsonism is associated with *PINK1* mutation [14]. Though it is well documented that alterations of lipid signalling and metabolism play a significant role in normal neuronal functions [15,16,17], little is known about the role and function of lipids associated with this progressive disorder of the nervous system. Increased levels of certain lipids within the substantia nigra (SN) of animal models of PD were demonstrated; lateral SN pars compacta was collected from rats 21 days after the infusion of 6-hydroxydopamine and two lipid species involved in neuroinflammatory signalling, namely lysophosphatidylcholines (16:0) and (18:1), were found up-regulated [18]. In another study carried out on the frontal cortex of PD subjects, selective decrements in the cortical levels of phosphatidylcholines were related to a concurrent increase in the pool size of free diglycerides [19]. Substantial changes in sphingolipid and glycerophospholipid biosynthetic pathways were revealed in the primary visual cortex, amygdala and anterior cingulate of human PD post-mortem brain tissues [20]. All these investigations, however, are performed on post-mortem biopsies reporting advanced neuronal damage so the early identification of neuronal changes represents an urgent and challenging task.

Human skin fibroblasts (FB) are an easily accessible source of proliferating cells that share the same genetic complexity of neurons and can reflect PD biomolecular dysfunctions [21,22,23]. Parkin, for instance, is expressed in human skin FB [24,25] and recent studies have demonstrated that primary FB of parkin-mutant patients, affected by early-onset recessive PD, displayed severe aberrations in mitochondria such as altered energy metabolism and amplified reactive oxygen species production [26,27]. A lipid characterization of these parkin-mutant FB has been recently attempted by thin layer chromatography (TLC) and off-line matrix assisted laser desorption ionization (MALDI) mass spectrometry (MS) [28]. Liquid chromatography (LC) coupled on line to high resolution MS is expected to outperform TLC off-line MALDI MS especially in highly complex samples [29,30].

Here, a LC-MS method based on hydrophilic interaction liquid chromatography (HILIC) coupled to electrospray ionisation-Fourier transform tandem mass spectrometry (ESI-FT MS/MS) is described for the identification of polar lipids occurring in human skin FB aimed at discovering potential biomarkers of PD. The proposed approach enabled the identification of 360 phospholipids (PL) belonging to various classes. By univariate analysis, a set of 61 PL were initially selected as significantly different in PD patients compared to the control one; most interestingly eight of them, namely PI 38:3, PC 34:1, PE 38:2, SM 36:2;2 and four plasmalogens, appeared down- or up-regulated according to the extent of disease progression as rated by the Hoehn and Yahr scale. These findings, although preliminary, suggest that the phospholipidome of human dermal FB represents a source of potential biomarkers for early diagnosis of Parkinson’s disease.

## 2. Results

### 2.1. Characterization of Phospholipids

**Figure 1:**
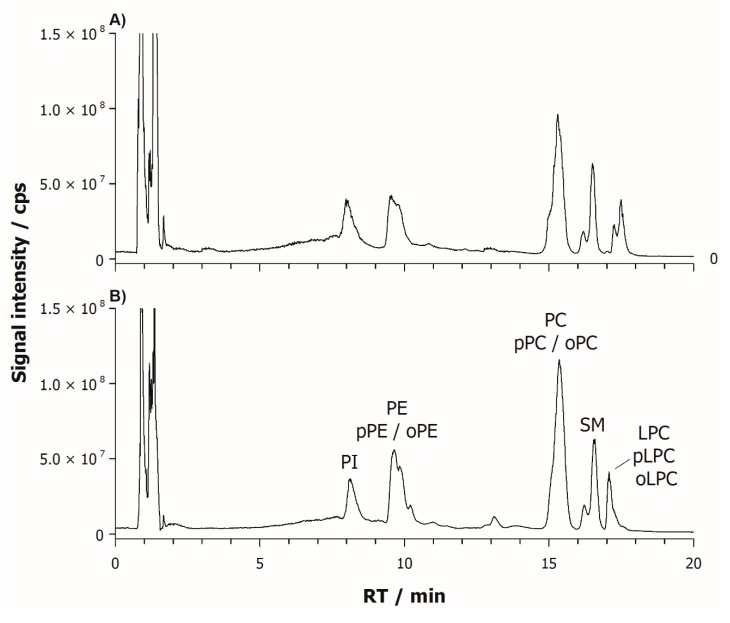
Total ion chromatograms (TICs) of human fibroblast lipid extract relevant to control cells (**A**) and Parkinson’s disease (PD) patient at Hoehn and Yahr (HY) stage 5 (**B**). TIC signals were acquired from three different independent replicates of each sample, aligned and averaged using MATLAB.

Recently, the separation of amphiphilic compounds including phospholipids in complex samples by HILIC-ESI-MS [29,30,31] has been proposed [32]. Figure 1 shows the total ion chromatograms (TIC) of FB lipid extracts in a control cellular line and in a patient affected by PD and classified at Hoehn and Yahr (HY) stage 5, plots (A) and (B), respectively.

**Figure 2:**
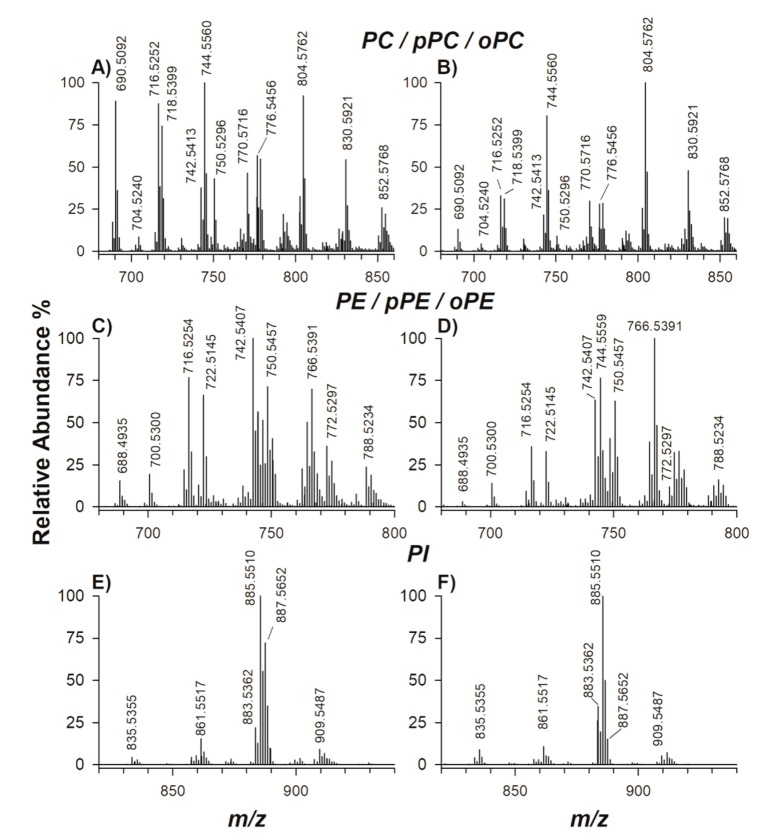
Fourier transform mass spectrometry (FTMS) spectra relevant to phosphatidylcholines (PC), phosphatidylethanolamines (PE) and phosphatidylinositols (PI) in negative ion mode averaged under the chromatographic bands of fibroblast lipid extract relevant to control cells (**A**,**C**,**E**) and PD patient with ex2del/ex4del deletion (**B**,**D**,**F**).

Each lipid class was separated within 20 min in an elution order which reflects the increasing headgroup polarity, i.e., phosphatidylinositols (PI), phosphatidylethanolamines (PE), phosphatidylserines (PS), lysophosphatidylethanolamines (LPE), phosphatidylcholines (PC) sphingomyelins (SM), and lysophosphatidylcholines (LPC). Mass spectra of major PC, PE and PI, mediated under the relevant chromatographic bands, are reported in plots (A,C,E) and (B,D,F) of Figure 2, for the control and PD patient, respectively. Source-induced dissociation [33] and negative ion mode operation provided useful information for the identification of PI, PE and LPE as deprotonated molecules [M − H]^−^, and PC, LPC and SM as demethylated molecules [M − CH_3_]^−^ and formate or acetate adducts (i.e., [M + HCOO]^−^, [M + CH_3_COO]^−^).

HILIC-ESI-FTMS analyses, complemented by all ion fragmentation (AIF) tandem MS experiments, performed at higher-energy collisional dissociation (HCD) regimes, provided the identification of 266 phospholipids: 104 PC, 28 PE, 14 LPC, 8 LPE, 84 PI and 28 SM (see Reference [32] for further details). Combining HCD and collisional induced dissociation (CID) regimes with tandem MS, phosphatidylserines (PS), eluting as a broad band from 13 to 20 min, and plasmalogens were additionally characterized including 59 PS, 17 plasmanyl-PC (1-alkyl-2-acyl-PC or oPC), 7 plasmanyl-PE (1-alkyl-2-acyl-PE or oPE) and 11 plasmenyl-PE (1-alkenyl-2-acyl-PE or pPE), see Table 1.

### 2.2. Control vs. PD Patients

As aforementioned, lipid extracts from FB samples of patients listed in Table 2 were examined and data compared with the control one. All the collected data were processed by Alex^123^ [34] tool searching for unambiguous assignments considering deprotonated signals of PS, PE, PI, LPE and oPE and demethylated adducts for PC, oPC, LPC, and SM with a tolerance of 0.005 *m/z* units.

Only lipids fully assigned by MS/MS data (see Table 1 and Reference [32]) and detected in all spectra were employed for ensuing statistical investigations. Note that the elution behaviour in HILIC of PS is more complex than other PL [35] resulting in a very broad band with low retention time reproducibility which hinders their accurate quantification. Therefore, these lipids, although possibly significant for their involvement in the PD, were not further processed. Even if LPE and LPC concentrations are often associated to different pathological conditions [36], it has been demonstrated that their levels are also strictly affected by a number of factors such as sample pre-treatment, temperature and time of sample storage [32]; for these reasons LPE, LPC and other lyso-forms were not further considered. In the searching for potential biomarkers, we were especially interested in changes in the lipid profiles, rather performing absolute quantification. Thus, the relative content of each lipid species was calculated by Alex^123^ software as the ratio between its peak intensity and the total peak intensities calculated for the whole mass spectrum of the class which the given lipid belongs to. Such an approach provides a sort of “internally normalized” data compensating for sample-to-sample analytical variability.

Normalized data obtained for the control sample (ctrl) were compared by a one-way ANOVA (*p* < 0.05) with data relevant the FB extract of the patient having the most severe PD stage (HY = 5, see Table 2). Notably, a total of 61 PL, namely 25 PC, 5 oPC/pPC, 11 PE, 8 oPE/pPE, 9 PI and 3 SM were found to change significantly in terms of relative signal intensity (see Table 3).

**Figure 3:**
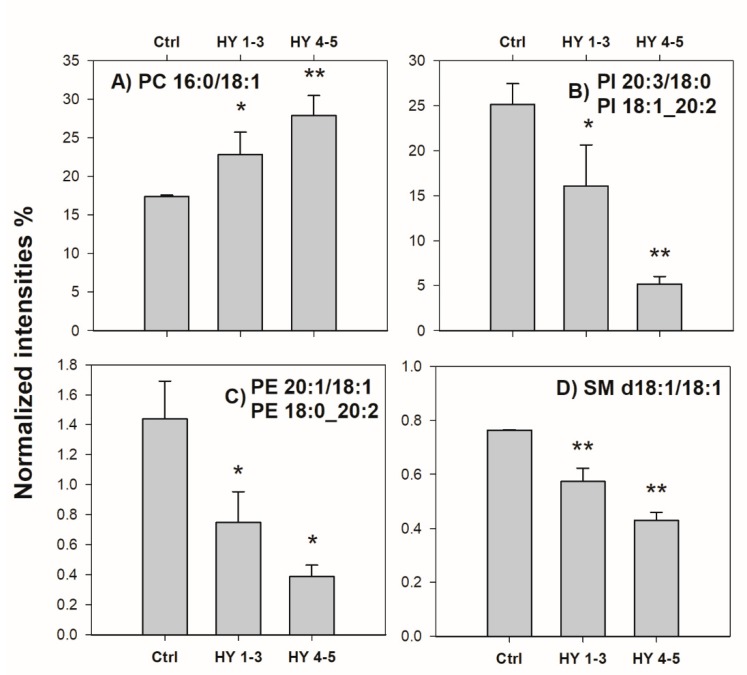
Bar plots of (**A**) PC (16:0/18:1), (**B**) PI (20:3/18:0) and (18:1_20:2), (**C**) PE (20:1/18:1) and (18:0_20:2), and (**D**) SM (d18:1/18:1) obtained as normalized intensities (as described in the text) plotted versus pathology degree specified as ctrl (control), group HY 1–3 and group HY 4–5 (see Table 2). Data are reported as average values on three independent replicates for each sample; error bars represent standard deviations. Bars marked with asterisks represent normalized intensities significantly different from those of control group (* *p* < 0.05; ** *p* < 0.01), as assessed using a nonparametric Welch’s two tails *t*-test.

Most interestingly, eight lipids belonging to this set appeared down- or up-regulated, depending on the HY stage of PD patients. Due to the limited number of patients enrolled in this preliminary study, two groups were generated: the first including two patients with HY stage 1 and 3, and the second one including three patients with HY stage 4 and 5. The significance of differences observed in lipid normalized intensities between each of the two groups and the control one was assessed using a nonparametric Welch’s two tails *t*-test, since population variances were unknown and different and sample sizes were small. As reported in Figure 3, PIs with overall side chain composition 38:3 (i.e., 20:3/18:0 and 18:1_20:2), PEs with composition 38:2 (i.e., 20:1/18:1 and 18:0_20:2) and SM d18:1/18:1 tended to be downregulated as the HY stage increased, whereas PC 16:0/18:1 tended to be up-regulated.

**Figure 4:**
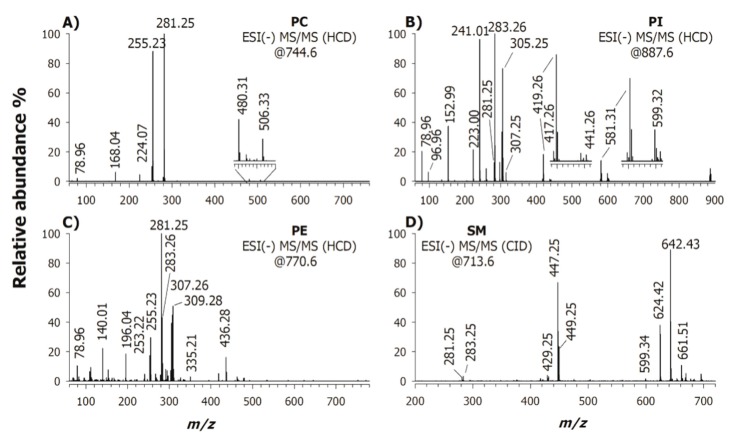
ESI(-)-MS/MS spectra of four glycerophospholipid putative biomarkers of PD (normalized collisional energy, 35%). (**A**) FTMS/MS spectrum of a PC at *m/z* 744.6 isolated as demethylated ion, [M − CH_3_]^−^, (**B**) FTMS/MS spectrum of a PI at *m/z* 887.6 isolated as deprotonated molecule [M − H]^−^, (**C**) FTMS/MS spectrum of a PE at *m/z* 770.6 as [M − H]^−^, and (**D**) linear ion trap (LIT) MS/MS spectrum of a SM at *m/z* 713.6 as [M − CH_3_]^−^.

Worth of note, some of those macroscopic variations can be simply retrieved just inspecting the high-resolution/accuracy MS spectra of Figure 2. Indeed, comparing plots E and F, signal at *m/z* 887.6 (i.e., PI 38:3) was significant lowered in the HY 5 PD patient, passing from 75% to the 20% of relative signal intensity. The mass spectrum inspection of PC cannot provide the desired information since one of the most abundant signals is due to PC 34:1 which is split into two forms, i.e., a demethylated molecule at *m/z* 744 and a formate adduct at *m/z* 804. Figure 4A reports the CID-MS/MS spectrum of PC (34:1) detected at *m/z* 744.6 as demethylated ion [M − 15]^−^ upon in-source methyl loss (CH_3_^+^) from the choline head group.

The signals detected at *m/z* 255.23 and 281.25 corresponded to carboxylate anions 16:0 (i.e., palmitic acid) and 18:1 (most likely oleic acid), respectively. Low intensity signals at *m/z* 480.31 ([M − CH_3_-264]^−^) and 506.33 ([M − CH_3_-238]^−^) refer to the loss as ketenes (i.e., [M − CH_3_ – R_1/2_CH=C=O]^−^) of the fatty acyl chains 18:1 and 16:0, whereas peaks at *m/z* 224.07, 168.04 and 78.96 were diagnostic fragments of phosphocholine polar head [37], i.e., [C_7_H_15_NO_5_P]^−^, N,N-dimethylphosphoethanolamine [C_4_H_11_NO_4_P]^−^ and monovalent metaphosphoric anion [PO_3_]^−^, respectively. Previously described interpretation rules [38] allowed to assign the regiochemistry as PC 16:0/18:1. Figure 4B shows the tandem mass spectrum of the precursor ion [M − H]^−^ at *m/z* 887.6 that, based on the accurate mass value, was preliminarily identified as PI 38:3. Note that the CID-MS/MS spectrum indicated the presence of two isobaric species (vide infra), i.e., PI (20:3/18:0) and (18:1_20:2) since the following peak signals were observed at *m/z* 281.25, 283.26, 305.25 and 307.25, referred to 18:1, 18:0, 20:3 and 20:2 carboxylate ions. Accordingly, a cluster of low intensity signals (inset of plot B in Figure 4) around *m/z* 581 and 599 validated the loss of the relevant acyl chain as fatty acids and as ketenes, respectively. These fragments can be involved in the polar head loss and the generation of another small group of signals around *m/z* 417–441. Typical PI product ions from the polar head at *m/z* 241.01, 223.00, 152.99, 96.96 and 78.96 were also revealed in the low mass range region. Since the remote charge-driven fragmentation processes is sterically more favourable at sn-2 over sn-1 [39], these isomers were assigned as PI 20:3/18:0 and PI 18:0_20:2. The MS/MS spectrum of the precursor ion at *m/z* 770.6, assigned as PE 38:2 by its accurate mass value, is displayed in Figure 4C. Again, following the established rules on regiochemistry [38], it was possible to state the presence of at least two isobaric species. In detail, the main product ions at *m/z* 281.25, 283.26, 307.26 and 309.28 allowed the assignment of two isobaric species as PE 20:1/18:1 and PE 20:2_18:0 while other low intensity peak signals at *m/z* 253.22, 255.23, 335.31 and 337.30 suggested the presence of PE 22:1/16:1 and PE 22:2/16:0.

The fragmentation mechanism of sphingomyelins (SM) differed from other PL because the backbone is a sphingoid base and the HCD-MS/MS fragmentation does not allow a straightforward assignment. Figure 4D is the CID tandem MS spectrum in negative ion mode of a SM (36:2;2) as [M − CH_3_]^−^ at *m/z* 713.6. The product ions related to the PC head group were those at *m/z* 642.43 and 624.42 corresponding to the neutral loss of choline moiety and choline plus water, respectively. The product ion at *m/z* 449.25 was due to the loss as ketene of the N-linked acyl chain allowing the identification of the sphingoid base (SB) as d18:1 [40,41]. Because of the occurrence of the carboxylate anion at *m/z* 281.25, such a species was thus recognized as SM d18:1/18:1.

**Figure 5:**
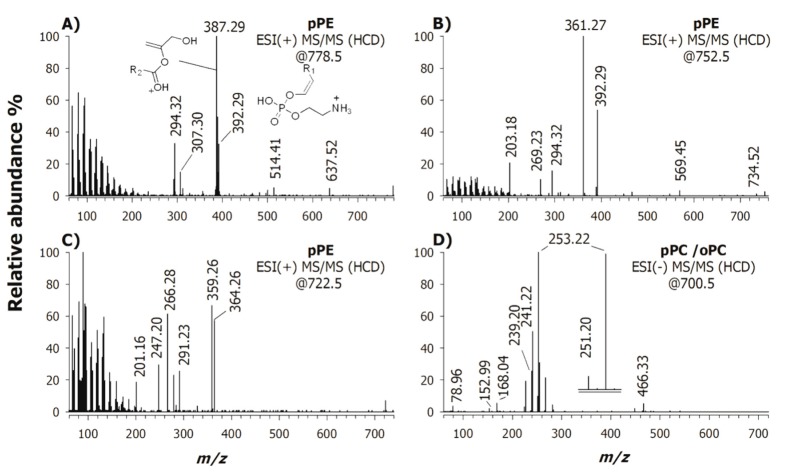
ESI-MS/MS (normalized collisional energy used: 35%) spectra obtained for the potential four plasmalogen biomarkers of PD pathology. ESI (+) FT-MS/MS spectra of (**A**) pPE at *m/z* 778.5, (**B**) *m/z* 752.5, (**C**) *m/z* 722.5, and (**D**) (−) FT-MS/MS spectrum of pPC at *m/z* 700.5.

The PE and PC classes were classified in three main groups: diacyl PE/PC, alkyl-acyl PE/PC (i.e., oPE or oPC) and alkenyl-acyl PE/PC (i.e., pPE or pPC); all these lipid species could be successfully examined by tandem MS. Plots (A), (B) and (C) of Figure 5 report HCD-MS/MS spectra of PE plasmalogens obtained in positive ion mode at m/z 778.5, 752.5 and 722.5, respectively; two prominent product ions, diagnostic of the acyl chains at sn-1 and sn-2 positions, are discernible [42]. Figure 5A shows that the ion with even *m/z* ratio 392.29 indicated a sn-1 alkenyl chain (p-18:0) whereas the most abundant ion with odd *m/z* ratio 387.29 designated the fatty acid in the sn-2 position (schematic structures are in the insets of Figure 5A). A low intensity signal at *m/z* 637.52 due to the loss of the phosphoethanolamine head group (141 Da) was diagnostic of a plasmalogen. The existence of an alkyl-acyl PE could be ruled out because in the case of oPE the product ion due to the neutral loss of ethanolamine (61 Da) should represent the base peak. Moreover, the product ion at *m/z* 292.32 was generated from the one including the alkenylic chain through subsequent loss of H_3_PO_4_, while product ions observed at lower *m/z* ratios were formed through consecutive fragmentation of the ion at *m/z* 387.29, containing the 22:5 acyl chain. The species under investigation was thus recognized as pPE (18:0/22:5). By following the same rule, we assigned the lipid species of plots (B) and (C) in Figure 5 as pPE (18:0/20:4) and pPE (16:0/20:5), respectively.

The peak signal occurring at *m/z* 700.5 was isolated and fragmented in negative ion mode since the accurate mass suggested a plausible plasmanyl PC species [43]. The tandem MS spectrum of the precursor ion [M − CH_3_]^−^ (*m/z* 700.5), reported in Figure 5D, showed abundant fragment ions at *m/z* 253.22, 251.20, 241.22, 239.20; the product ion at *m/z* 241.22 was supposed the alkyl ion identifying the chain located at the sn-1 position of glycerol in conjunction with the product ion at *m/z* 251.20 relevant to the 16:2 fatty acid, thus suggesting a plasmanyl PC with regiochemical composition, oPC 16:0/16:2. Yet, also an alkenyl ion was detected at *m/z* 239.20 likely associated with the product ion at *m/z* 253.22, thus signifying the presence of the isobaric species, i.e., pPC 16:0/16:1. In the low mass range, the absence of a peak at *m/z* 224.07 arising, for PC and LPC, from the loss of the acyl chain(s), as fatty acid and/or as ketene confirmed the assignment as plasmanyl- or plasmenyl-PC. Additional peak signals were detected at *m/z* 168.04, corresponding to the deprotonated form of N,N-dimethylphosphoethanolamine, and at *m/z* 152.99, which was the glycerol-3-phosphate minus water and [PO_3_]^−^ at *m/z* 78.96.

**Figure 6:**
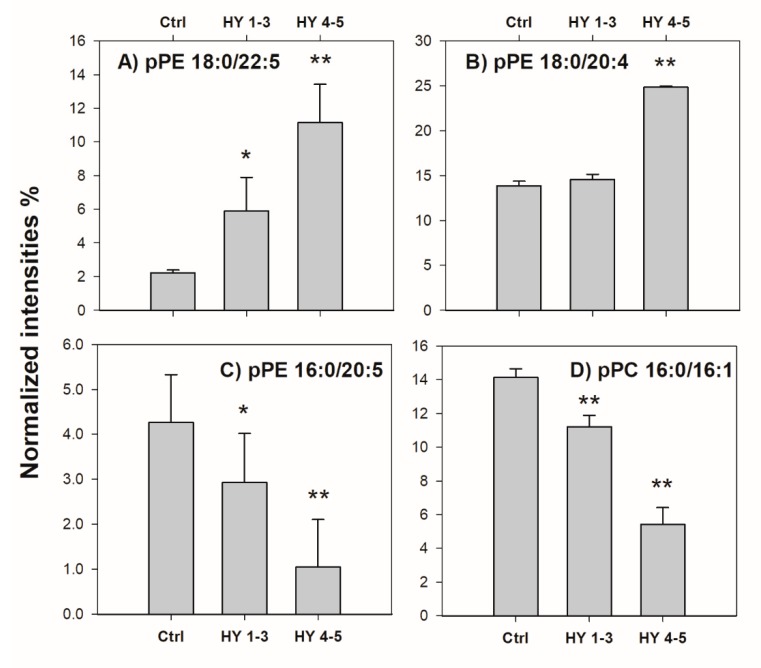
Bar plots of (**A**) pPE (18:0/22:5) at *m/z* 776.5, (**B**) pPE (18:0/20:4) at *m/z* 750.5, (**C**) pPE (16:0/20:5) at *m/z* 720.5 and **D**) pPC (16:0/16:1) at *m/z* 700.5 obtained as normalized intensities (as described in the text) plotted versus pathology degree specified as ctrl (control), group HY 1-3 and group HY 4-5 (see Table 2). Data are reported as average values on three independent replicates for each sample; error bars represent standard deviations. Bars marked with asterisks represent normalized intensities significantly different from those of the control group (* *p* < 0.05; ** *p* < 0.01), as assessed using a nonparametric Welch’s two tails *t*-test.

By examining the relative content of all PL identified in FB lipid samples, we found evidence that four plasmalogens (i.e., pPE (18:0/22:5), pPE (18:0/20:4), pPE (16:0/20:5) and pPC (16:0/16:1)) were up- or down-regulated to an extent reflecting the progression of PD. This outcome is illustrated in Figure 6 (the same approach adopted for data reported in Figure 3 was used). Except pPE (18:0/20:4), the other three PL species appeared correlated with the PD progression, a highly desirable feature of a diagnostic biomarker for use in the clinical and preclinical stages of PD.

## 3. Discussion

The importance of the amounts and types of lipids forming a membrane, in controlling and preserving the biological functions, is well recognized. A range of inherited disorders are associated with PL catabolism. In the case of PD, the role of lipids has been recently reviewed [44]; while it is clear that a strong correlation exists between impairment of neural functions and lipid accumulation, the available literature information is fragmented and often not reproducible since likely influenced by sex, age, PD etiology, DNA polymorphism and microbiome of the patients. Most of the existing lipidomic studies are performed on easily accessible plasma samples and a lack of correlation with lipid levels in cerebrospinal fluid and/or brain cannot be ruled out. This implies that the identification and validation of lipid biomarkers in circulating biological fluids are very challenging tasks. Lipidomics of skin fibroblasts, used as model systems for PD, is still in its infancy. A study of Valsecchi et al. [45] reported on the content of different species of Ceramide (cer) and sphingomyelin (SM) in cell homogenates from fibroblasts and neurons in culture analysed by mass spectrometry. In particular, they focused on the distribution of sphingolipids and phosphatidylethanolamines (PE) within detergent-soluble fraction and detergent-resistant membranes (DRM) prepared from cultures of human skin fibroblasts and primary cultured rat neurons. Comparing results, they found that glycolipids and SM were highly enriched in DRMs from both cell systems and the content of ceramides was lower in DRMs from fibroblasts than neurons. Other studies also reported correlation between lipids in fibroblasts and neurons even if a quantitative analysis is not performed [46,47]. A limited set of lipids have been characterized in skin fibroblasts of parkin-mutant patients by MALDI MS [28], whereas a more extensive, but still not exhaustive, lipidome characterization by HILIC ESI FTMS has been undertaken in our laboratory [32]. These recently reported data along with those here described lead to the identification of 360 phospholipids.

When working with lipidomic datasets consisting of hundreds of parameters as the lipids identified in many samples, a major challenge is to extract the appropriate information. In this context the relevant information is related to the lipids that discriminate between a cohort of healthy versus diseased subjects. The analysis of large datasets is the trickiest endeavour and the support of statistics aiming at the identification of biomarkers is fundamental to interpret complex data sets. Here by applying the parametric *t*-test, 61 lipids were found dysregulated when the control was compared with the patient rated as 5 on the HY scale. Some of these species (e.g., PS 36:1; PI 38:4) have been already reported in other studies [28], while altered levels of total SM have been reported in plasma of PD patients with glucosylceramidase beta (*GBA*) mutation [48]; SM 18:1 and SM 26:1 were increased and decreased in the anterior cingulate cortex [49], respectively, and increased SM levels were described in the primary visual cortex of PD patients [20]. However, the unprecedent finding here reported was the identification of eight lipids (see Figure 3 and Figure 6) reflecting the PD progression whereby four of them were PE or PC plasmalogens with long-chain polyunsaturated fatty acids (PUFA). Plasmalogens PE represent a definite PL class playing critical roles in membrane functions, vesicular release of neurotransmitters and free radical scavenging [50]. It is known that plasmalogens constitute about 30 mol% of the total brain phospholipids and about 70% of PL in myelin [50]. The altered level of plasmalogens is in general agreement with the conclusions reported in previous studies where a decrement of pPE was observed in brain tissue and cerebrospinal fluid of Alzheimer’s disease patients [51,52]. Plasmalogens, as well, are recognized as important markers of oxidative stress conditions related to many human dysfunctions; they are considered as sacrificial oxidants since they are preferentially oxidized when exposed to free radicals, due to the presence of the hydrogen atoms adjacent to the vinyl ether bond which show relatively low disassociation energies [53]. Besides, plasmalogens are required for the correct function of integral membrane proteins and for the generation of lipid second messengers [50]. It has also been reported that PD patients had significantly decreased levels of ether-linked lipids of frontal cortex lipid rafts [52]. Usually, ether linked lipids are known to be platelet activating factors and have been shown to be heavily involved in the neuroinflammatory responses [50]; as other neurodegenerative disorders, PD is also recognized to have an inflammatory component. Therefore, the eight PL identified in this preliminary study, i.e., PC (16:0/18:1), PI (20:3/18:0), PE (20:1/18:1), SM (d18:1/18:1), pPE (18:0/22:5), pPE (18:0/20:4), pPE (16:0/20:5) and pPC (16:0/16:1), represent a set of putative biomarkers deserving further investigation and validation. These potential indicative biomarkers should facilitate the diagnosis in the clinical and especially preclinical stages of PD.

## 4. Materials and Methods

### 4.1. Chemicals

Water, acetonitrile, methanol, chloroform, formic acid and ammonium acetate were obtained from Sigma-Aldrich (Milan, Italy). Standard lipids were purchased from Spectra 2000 SRL (Rome, Italy). Age-matched adult normal human dermal fibroblasts (NHDF) were purchased from Lonza Walkersville Inc., (Walkersville, MD, USA). All solvents used were LC–MS grade except for CHCl_3_ (HPLC grade). A calibrating solution containing caffeine, methionine–arginine–phenylalanine–alanine peptide and Ultramark, a mixture of fluorinated phosphazines, for positive and negative calibrations were purchased from Thermo Scientific (Waltham, MA, USA). The lipid nomenclature described by Liebisch et al. [54,55] was adopted throughout this paper. Briefly, acyl chain compositions are indicated by the number of carbon atoms in the acyl chain followed by a colon and the number of double bonds (e.g., C:D).

### 4.2. Sample Preparation

#### 4.2.1. Samples and Cell Growth

Five patients showing an early onset form of PD were selected for this study (Table 2). Diagnosis of PD was made according with the UK Brain Bank criteria: patients underwent neurological examination including the Unified Parkinson’s Disease Rating Scale (UPDRS) and Hoehn and Yahr scale. The clinical phenotype was characterized by good response to levodopa, early fluctuation, dyskinesia and psychiatric symptoms. Primary skin fibroblasts were obtained by explants from skin punch biopsy. Skin biopsies were obtained after informed consent; age-matched adult normal human dermal fibroblasts (NHDF) were used as healthy control. Cells were grown in high-glucose Dulbecco’s modified Eagle’s medium (DMEM) supplemented with 10% (*v*/*v*) fetal bovine serum (FBS), 1% (*v*/*v*) l-glutamine, 1% (*v*/*v*) penicillin/streptomycin, at 37 °C in a 95% humidified atmosphere. All experiments were performed on cells with equal passage numbers, ranging from 5 to 10, to avoid an artefact due to senescence, known to occur at passage numbers greater than 30. After reaching a 70–80% confluence, the cells were washed with phosphate buffer saline (PBS) medium and detached from the Petri dish using a solution containing 0.05% trypsin and 0.2% EDTA. The cells were centrifuged at 500× *g* for 4 min; the pellet was resuspended in PBS for successive lipid extraction.

#### 4.2.2. Lipid Extraction

Following the Bligh and Dyer protocol [56], 3 mL of methanol/chloroform (2:1, *v*/*v*) were added to 50 µL (5 × 10^7^ cells) of fibroblast (FB) sample homogenate diluted with 750 µL of water and the mixture was left for one hour at room temperature. Then, 1 mL of chloroform was added, and the mixture was vortexed for 30 s. Finally, 1 mL of water was added, and the solution was shaken before being centrifuged for 10 min at 3000× *g*. The lower phase, containing lipids, was dried under nitrogen and then redissolved in 50 µL of methanol and analysed by LC-MS. The lipid/protein ratio was about 0.2 (*w*/*w*) in the cell lines here considered.

### 4.3. HILIC-ESI-MS Instrumentation and Operating Conditions

HILIC-ESI-FTMS measurements were performed using an LC-MS apparatus consisting of an Ultimate 3000 UHPLC system and a hybrid Q-Exactive mass spectrometer (Thermo Scientific, Waltham, MA, USA), equipped with a heated electrospray ionization (HESI) source and a higher collisional energy dissociation (HCD) cell for tandem MS analyses. Chromatographic separations were run at ambient temperature on a narrow-bore Ascentis Express HILIC column (150 × 2.1 mm ID, 2.7 μm particle size) equipped with an Ascentis Express HILIC (5 × 2.1 mm ID) security guard cartridge (Supelco, Bellefonte, PA, USA) using a flow rate of 0.3 mL·min^−1^. A volume of 5 μL of lipid extract was injected into the column using a RS Autosampler (Thermo Scientific, Waltham, MA, USA). The adjusted binary elution program, based on water and 2.5 mmol·L^−1^ ammonium formate (solvent A) and acetonitrile (solvent B), both containing 0.1% (*v*/*v*) of formic acid, was adopted: 0–5 min, linear from 97% to 88% solvent B; 5–10 min, isocratic at 88% solvent B; 10–11 min, linear from 88% to 81% solvent B; 11–20 min, linear gradient from 81% to 70% solvent B; 20–22 min, linear from 70% to 50% solvent B; 22–28 isocratic at 50% solvent B; 28–30 min, return to the initial composition, followed by a 5 min equilibration time.

The column effluent was transferred into the Q Exactive spectrometer through the HESI source. The main ESI and ion optic parameters were the following: sheath gas flow rate, 35 (arbitrary units); auxiliary gas flow rate, 15 (arbitrary units); spray voltage, 3.5 kV (positive) and −2.5 kV in negative polarity; capillary temperature, 320 °C; S-lens radio frequency level, 100 (arbitrary units). Negative and positive MS full-scan spectra were acquired in the *m/z* range 130–2000, after setting a mass resolving power of 140,000 (at *m/z* 200). During MS measurements, the Orbitrap fill time was set to 200 ms and the automatic gain control (AGC) level to 3 × 10^6^. The Q Exactive spectrometer was daily calibrated and mass accuracies ranged between 0.10 and 0.17 ppm in positive polarity and between 0.40 and 0.45 ppm in negative polarity.

Definite assignment of each lipid species was obtained performing further LC-MS runs using targeted-MS^2^ acquisitions. In this case, the exact *m/z* values of the selected precursor ions were introduced into an inclusion list, each with a tolerance of 10 ppm. MS/MS measurements were performed, in both positive and negative polarities, using a 1 *m/z* unit wide window, a resolving power of 70,000 (at *m/z* 200), a fill time of 100 ms and AGC of 2 × 10^5^. Further HILIC-MS measurements were performed in parallel using a medium resolving power and mass accuracy apparatus (Velos Pro mass spectrometer—Thermo Scientific) equipped with a linear ion trap (LIT) analyser and a HESI interface. The double-stage LIT mass analyser working in a low-energy collisionally induced dissociation (CID) regime was complementary to HCD to confirm some doubtful attributions of minor species. Only the S-lens radio frequency level, lowered to 60 (arbitrary units), was modified among HESI and ion optic parameters when using the Velos Pro spectrometer. The control of the LC-MS instrumentation and the first processing of data were performed by the Xcalibur software, version 3.0.63 (Thermo Scientific). The post analyses data processing was performed by using SigmaPlot 11.0 to graph final mass spectra.

### 4.4. Data Treatment

Each phospholipid extract was analysed in triplicate. Mass spectra, averaged under HILIC chromatographic band, were analysed by Alex^123^ data search tool, employing target lists generated using demethylated PC, oPC, SM and LPC and deprotonated PE, oPE, PS and PI with 0.005 *m/z* tolerance. MS/MS analyses performed on a pooled sample were used to confirm putative attributions, and only characterized lipids found in all replicates were used for statistical analysis after lipid class normalization. Identification of potential biomarkers related to Parkinson disease was accomplished by *t*-test comparing the means of each variable (normalized data) between the most severe Hoehn and Yahr scale stage patient (HY 5) and control. For statistics, MATLAB software was employed (version 2018, mathworks); the level of significance was set at *p* = 0.05.

## 5. Conclusions

The phospholipidome of human dermal fibroblasts has been characterized by HILIC-ESI-MS. Up to 360 phospholipids, differing in head groups, backbones, and fatty acyl moieties, were identified. The phospholipidome of FB obtained from skin biopsies of five patients with Parkinson’s disease at different progression stages (Hoehn and Yahr scale) was compared to a control. This is a step forward in demonstrating that parkin-mutant fibroblast cultures can mirror lipidomic changes involved in neurological disorders thus offering easily accessible biological specimen replacing post-mortem brain tissues. The most interesting finding is represented by the evidence that eight PL (including four plasmalogens) out of the 61 lipids set above mentioned, were up- or down-regulated to an extent reflecting the progression of PD. Such lipids could be proposed as potential biomarkers, though additional validation is deserved by enrolling a larger number of healthy control and PD patients. Further studies are also underway to characterize other minor lipid compounds in fibroblasts which could be eventually correlated to PD disease [57].

## Figures and Tables

**Table 1:** Minor phospholipids identified in human dermal fibroblasts by liquid chromatography electrospray ionisation tandem mass spectrometry (LC-ESI(−) MS/MS) and mass spectrometry (MS)^3^.

Experimental Value (*m/z*)	Adduct	Regiochemical Assignment	Molecular Formula (M)
***Plasmenyl-phosphatidylcholine*** **(*p-PC*)**			
700.5286	[M − CH_3_ − H]^−^	(16:0/16:1)	C_39_H_75_NO_7_P
702.5448	[M − CH_3_ − H]^−^	(16:0/16:0)	C_39_H_77_NO_7_P
***Plasmanyl-phosphatidylcholine*** **(*o-PC*)**			
700.5286	[M − CH_3_ − H]^−^	(16:0/16:2)	C_39_H_75_NO_7_P
702.5448	[M − CH_3_ − H]^−^	(16:0/16:1); (15:0_17:1)	C_39_H_77_NO_7_P
714.5443	[M − CH_3_ − H]^−^	(16:1_19:1)	C_40_H_77_NO_7_P
716.599	[M − CH_3_ − H]^−^	(18:1/15:0); (16:1/17:0)	C_40_H_79_NO_7_P
718.5756	[M − CH_3_ − H]^−^	(17:0_16:0)	C_40_H_81_NO_7_P
732.5912	[M − CH_3_ − H]^−^	(16:0/18:0); (18:0/16:0); (17:0/17:0)	C_41_H_83_NO_7_P
752.5599	[M − CH_3_ − H]^−^	(16:0/20:4)	C_43_H_79_NO_7_P
756.5912	[M − CH_3_ − H]^−^	(16:0/20:2)	C_43_H_83_NO_7_P
762.5654	[M + HCOO]^−^	(16:0/16:1)	C_41_H_81_NO_9_P
780.5912	[M − CH_3_ − H]^−^	(18:0/20:4)	C_45_H_83_NO_7_P
802.5967	[M + HCOO]^−^	(17:1/18:1)	C_44_H_85_NO_9_P
812.5811	[M + HCOO]^−^	(16:0/20:4)	C_45_H_83_NO_9_P
***Plasmenyl-phosphatidylethanolamine*** **(*p-PE*)**			
672.4973	[M − H]^−^	(16:0/16:1)	C_37_H_71_NO_7_P
698.5130	[M − H]^−^	(16:0/18:2)	C_39_H_73_NO_7_P
700.5286	[M − H]^−^	(16:0/18:1)	C_39_H_75_NO_7_P
720.4973	[M − H]^−^	(16:0/20:5)	C_41_H_71_NO_7_P
722.5130	[M − H]^−^	(16:1/20:4)	C_41_H_73_NO_7_P
728.5599	[M − H]^−^	(18:0/18:1)	C_41_H_79_NO_7_P
748.5286	[M − H]^−^	(18:1/20:4)	C_43_H_75_NO_7_P
750.5443	[M − H]^−^	(18:0/20:4)	C_43_H_77_NO_7_P
772.5286	[M − H]^−^	(18:1/22:6)	C_45_H_75_NO_7_P
774.5442	[M − H]^−^	(18:1/22:5)	C_45_H_77_NO_7_P
776.5599	[M − H]^−^	(18:0/22:5)	C_45_H_79_NO_7_P
***Plasmanyl-phosphatidylethanolamine*** **(*o-PE*)**			
702.5443	[M − H]^−^	(16:0/18:1)	C_39_H_77_NO_7_P
716.5588	[M − H]^−^	(17:0/18:2)	C_40_H_77_NO_7_P
728.5599	[M − H]^−^	(16:0/20:2)	C_41_H_79_NO_7_P
762.5443	[M − H]^−^	(17:0/22:5)	C_44_H_79_NO_7_P
764.5599	[M − H]^−^	(17:0/22:5) (19:0/20:5)	C_44_H_79_NO_7_P
776.5599	[M − H]^−^	(18:0/22:6)	C_45_H_79_NO_7_P
***Phosphatidylserine*** **(*PS*)**			
700.5304	[M − H]^−^	(18:1/12:2); (18:2/12:1)	C_36_H_63_NO_10_P
702.5433	[M − H]^−^	(18:0/12:2); (14:1/16:1)	C_36_H_65_NO_10_P
732.482	[M − H]^−^	(16:0/16:1); (18:1_14:0)	C_38_H_71_NO_10_P
734.498	[M − H]^−^	(16:1/16:1)	C_38_H_73_NO_10_P
756.4820	[M − H]^−^	(18:2_16:1); (18:1_16:2)	C_40_H_71_NO_10_P
758.4980	[M − H]^−^	(16:0/18:2); (18:1/16:1)	C_40_H_73_NO_10_P
760.5149	[M − H]^−^	16:0/18:1; 16:1_18:0	C_40_H_75_NO_10_P
774.5303	[M − H]^−^	(18:0/17:1); (18:1_17:0); (16:0_19:1); (16:1_19:0)	C_41_H_77_NO_10_P
782.4977	[M − H]^−^	(16:0/20:4); (18:2_18:2)	C_42_H_73_NO_10_P
784.5134	[M − H]^−^	(16:0/20:4); (18:1_18:3)	C_42_H_75_NO_10_P
786.5309	[M − H]^−^	(18:0/18:2); (18:1/18:1); (16:0/20:2); (16:1_20:1)	C_42_H_77_NO_10_P
788.4977	[M − H]^−^	(18:0/18:1); (16:0_20:1)	C_42_H_79_NO_10_P
790.5532	[M − H]^−^	(18:0/18:0)	C_42_H_81_NO_10_P
796.5134	[M − H]^−^	(17:0/20:4)	C_43_H_75_NO_10_P
800.5447	[M − H]^−^	(19:1_18:1)	C_43_H_75_NO_10_P
806.4977	[M − H]^−^	(16:0/22:6); (18:1_20:5)	C_44_H_73_NO_10_P
808.5134	[M − H]^−^	(16:0/22:5); (18:1_20:4); (18:0_20:5)	C_44_H_75_NO_10_P
810.5297	[M − H]^−^	(18:1/20:3); (18:0_20:4)	C_44_H_77_NO_10_P
812.5450	[M − H]^−^	(18:0/20:3); (18:1_20:2)	C_44_H_79_NO_10_P
816.5760	[M − H]^−^	(18:0/20:1); (18:1_20:0)	C_44_H81NO_10_P
826.5603	[M − H]^−^	(18:0/21:3)	C_45_H_81_NO_10_P
832.5130	[M − H]^−^	(18:0/22:7); (18:1_22:6)	C_46_H_75_NO_10_P
834.5290	[M − H]^−^	(18:0/22:6); (20:4_20:2)	C_46_H_77_NO_10_P
836.5450	[M − H]^−^	(18:0/22:5); (18:1_22:4); (20:1/20:4)	C_46_H_79_NO_10_P
838.5603	[M − H]^−^	(18:0_22:4)	C_46_H_81_NO_10_P
840.5760	[M − H]^−^	(18:0/22:3)	C_46_H_83_NO_10_P
844.6070	[M − H]^−^	(18:0/22:1); (16:0_24:1); (18:1_22:0)	C_46_H_87_NO_10_P
856.5134	[M − H]^−^	(20:3_22:6)	C_48_H_75_NO_10_P
858.5290	[M − H]^−^	(20:3_22:5)	C_48_H_77_NO_10_P
860.5450	[M − H]^−^	(20:0_22:7); (20:1/22:6)	C_48_H_79_NO_10_P

Plasmalogen lipids are divided into plasmanyl-phospholipids (o-PL) which have an ether bond in position sn-1 to an alkyl group, and plasmenyl-phospholipids (p-PL) with an ether bond in position sn-1 to an alkenyl group. Legend: “/”: Fatty acyl chains positional isomeric level known; “_”: Regiochemistry not univocally established.

**Table 2:** Sex, age at skin biopsy, age at PD onset, genotypic characteristics and disease severity following the Hoehn and Yahr (HY) scale for five patients enrolled in this study. The control is a 53 years old female.

Patient	Sex	Age at Skin Biopsy (yr)	Age at PD Onset (yr)	PARK2 Mutation	HY Stage
**#1**	F	36	28	p.Cys253Tyr */ex5del	1
**#2**	M	64	47	ex3-4del/ex3-4del	3
**#3**	M	38	18	ex2-3del/ex2del	4
**#4**	M	65	33	ex2del/ex2-4del	4
**#5**	M	62	20	ex2del/ex2-4del	5

* Unclear pathogenic nature.

**Table 3:** Summary of lipids down- or up-regulated (compared to a control) in a PD patient rated as 5 on the Hoehn and Yahr scale.

PC	oPC/pPC	PE	oPE/pPE	PI	SM
31:2 ↑ 33:0 ↑ 33:1 ↑ 33:2 ↑ 34:0 ↑ 34:1 ↑ 34:4 ↑ 35:1 ↑ 35:2 ↑ 36:1 ↑ 36:2 ↑ 37:1 ↑ 38:4 ↑ 40:5 ↑ 40:6 ↑ 28:0 ↓ 28:1 ↓ 29:1 ↓ 30:0 ↓ 30:1 ↓ 32:1 ↓ 32:2 ↓ 36:4 ↓ 38:2 ↓ 39:3 ↓	34:0 ↑ 36:4 ↑ 38:4 ↑ 32:1 ↓ 32:2 ↓	36:1 ↑ 37:4 ↑ 38:4 ↑ 40:5 ↑ 32:1 ↓ 36:2 ↓ 36:4 ↓ 36:5 ↓ 38:2 ↓ 38:3 ↓ 38:6 ↓	38:5 ↑ 39:5 ↑ 39:6 ↑ 40:6 ↑ 32:2 ↓ 34:3 ↓ 36:6 ↓ 38:6 ↓	35:1 ↑ 35:2 ↑ 38:4 ↑ 38:5 ↑ 40:4 ↑ 40:5 ↑ 37:3 ↓ 38:3 ↓ 39:4 ↓	34:0;2 ↑ 35:1;2 ↑ 36:2:2 ↓

Legend: (↓) downregulated; (↑) upregulated. PC: phosphatidylcholine, oPC: plasmanyl-phosphatidylcholine, pPC: plasmenyl-phosphatidylcholine, PE: phosphatidylethanolamine oPE: plasmanyl-phosphatidylethanolamine, pPE: plasmenyl-phosphatidylethanolamine, PI: phosphatidylinositol, SM: sphingomyelin.
